# Immunity, Sex Hormones, and Environmental Factors as Determinants of COVID-19 Disparity in Women

**DOI:** 10.3389/fimmu.2021.680845

**Published:** 2021-08-18

**Authors:** Suriya Rehman, Vijaya Ravinayagam, Insha Nahvi, Hanan Aldossary, Maha Al-Shammari, Mai Saad Al Amiri, Uday Kishore, Ebtesam A. Al-Suhaimi

**Affiliations:** ^1^Department of Epidemic Disease Research, Institute of Research and Medical Consultations (IRMC), Imam Abdulrahman Bin Faisal University, Dammam, Saudi Arabia; ^2^Deanship of Scientific Research and Institute of Research and Medical Consultations (IRMC), Imam Abdulrahman Bin Faisal University, Dammam, Saudi Arabia; ^3^Department of Basic Sciences, Preparatory Year Deanship, King Faisal University, Al Hofuf, Saudi Arabia; ^4^Department of Public Health, Institute of Research and Medical Consultations (IRMC), Imam Abdulrahman Bin Faisal University, Dammam, Saudi Arabia; ^5^Department of Obstetrics and Gynecology, Maternity and Children Hospital, Dammam, Saudi Arabia; ^6^Biosciences, College of Health, Medicine and Life Sciences, Brunel University London, Uxbridge, United Kingdom; ^7^Biology Department, College of Science and Institute of Research and Medical Consultations (IRMC), Imam Abdulrahman Bin Faisal University, Dammam, Saudi Arabia

**Keywords:** COVID-19, environment, estrogen, immunity, gender, hormones, microbiota

## Abstract

The current coronavirus disease 2019 (COVID-19), caused by severe acute respiratory syndrome virus 2 (SARS-CoV-2), has resulted in a major global pandemic, causing extreme morbidity and mortality. Few studies appear to suggest a significant impact of gender in morbidity and mortality, where men are reported at a higher risk than women. The infectivity, transmissibility, and varying degree of disease manifestation (mild, modest, and severe) in population studies reinforce the importance of a number of genetic and epigenetic factors, in the context of immune response and gender. The present review dwells on several contributing factors such as a stronger innate immune response, estrogen, angiotensin-converting enzyme 2 gene, and microbiota, which impart greater resistance to the SARS-CoV-2 infection and disease progression in women. In addition, the underlying importance of associated microbiota and certain environmental factors in gender-based disparity pertaining to the mortality and morbidity due to COVID-19 in women has also been addressed.

## Introduction

The coronaviruses belong to the subfamily *Coronavirinae*, which cause respiratory and gastrointestinal infections (1). First discovered in 1960, the coronaviruses were ascribed to causing a mild respiratory symptom; these viruses include human CoV 229E (HCoV-229E) and HCoV-OC43 (2). The present coronavirus disease 2019 (COVID-19) pandemic by severe acute respiratory syndrome virus 2 (SARS-CoV-2) initially emerged from Wuhan Province, China at the seafood market (3). Various studies on the innate and adaptive immune responses to coronaviruses have been carried out in recent years. The role of the immune responses is to initiate viral clearance, prevent viral replication, and help tissue repair. However, such immune responses play a crucial part in SARS-related pathogenicity. The SARS-CoV-2 is known to dysregulate cytokine-mediated inflammatory and immune responses (4). Innate immune humoral factors such as complement and coagulation-fibrinolysis systems, soluble proteins/naturally occurring antibodies, and cellular components (natural killer cells and other innate lymphocytes) seem to be fully engaged following viral infection. Dysregulation of these factors leads to viral replication in the lung airways and escalation of an adaptive immune response. Severity caused by SARS-CoV-2 infection thus may also be attributed to the degree of dysregulated immune and inflammatory response (5).

**Figure 1:**
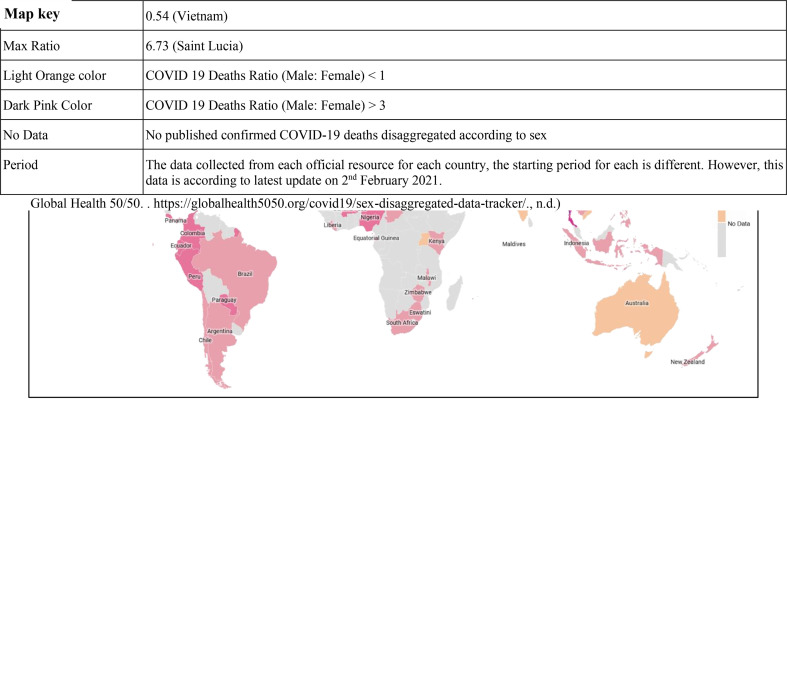
Global map showing the confirmed COVID-19 death incidence (male and female ratio) in various countries.

The virus has affected the global population; however, men seem to manifest more severe form of the disease than women, as per the onset of symptoms of the disease. The mortality in men is 2.4 times compared to women, although both gender have a similar susceptibility to transmission (6). One study that involved 425 COVID-19 patients reported 56% men (7), while another study reported 50.7% of 140 patients being men as infected individuals (8). Another study involving 1,019 COVID-19 patients revealed greater susceptibility of men compared to women to SARS-CoV-2, indicating that gender is as a risk factor for morbidity and mortality (6). One of the most noticeable differences is the mortality rates among men and women in the Western Europe, where 69% of men have died due to COVID-19. Even in the United States of America, a lesser percentage of women have died as compared to men (9). Similar patterns have been seen in China and other affected countries. According to one of the reports, the greatest sex disparity was seen in the death rate; it came to only 36.2% deaths in women as compared to men at the rate of 51.4%. Additionally, the analysis of COVID-19 cases documented in China showed a 2.8% case fatality in men as compared with a 1.7% rate in infected women (10) (**Figure 1**).

This is not the first time that coronaviruses have been found to affect women lesser than men. The epidemiological data from SARS-CoV (2003) and MERS-CoV (2012) epidemics also highlighted women at a lower risk of death from these deadly viruses (11). In Hong Kong, men were found to be affected more severely than women by the SARS-CoV (12). Furthermore, men had a significantly higher fatality rate than women (21.9% *versus* 13.2%) (13). In 2012, when MERS-CoV hit Saudi Arabia, the disease occurrence among men (62%) was considerably higher than in women (38%) of the total confirmed infected cases (14). Thus, gender seems to play an important role in severity and fatality in SARS-related diseases.

## Estrogen Acts as an Immune-Stimulating Factor

As men are worse affected by SARS-CoV-2, they require longer hospital stay and have a higher mortality rate when compared to women (15). The observed resistance to SARS-CoV-2 in women can be attributed to sex hormones, specifically estrogen, which is known to enhance the immune activity of both B as well as T-helper cells (16). Estrogen receptor alpha (ERα) is a steroid hormone receptor that controls physiological functions, including immunity. ERα has an effect on the subsets of T cells that includes Th1, Th2, Th17, and T regulatory cells, as well as follicular helper T (TFH) cells. It has been established that induced immunization by NP-conjugated ovalbumin produces specific antibodies that are elevated in CD4-ERα knock-out mice, under sufficient estrogen environment (17). Therefore, estrogen, the primary female sex hormone, stands out as a key biological factor making women’s immune system more active against the virus (13).

There is a growing interest in studying the role of sex hormones in the tissue renin-angiotensin system (RAS). The expression of ACE2 (angiotensin-converting enzyme) in some organs, such as uterus, kidney, and heart, is regulated by 17β-estradiol. This occurs by increasing the locally existing ACE2 effect on the cardiac tissue and suppressing the RAS through catalytic cleavage of a particular residue of angiotensin II to increase the release of cardioprotective angiotensin 1–7 and upregulate anti-oxidative and anti-inflammatory effects (18) Estrogen level is inversely related with the regulation of cardiac troponin secreted during ischemic or anoxic condition, leading to irreversible injury to the cardiac cells (19). In few studies conducted on COVID-19 patients, it was seen that 51% patients died due to cardiac injury (20). The death rate in COVID-19 patients was 7.6% having normal cardiac troponin levels and without any cardiovascular disease. Mortality of 13.3% was seen in patients with underlying cardiovascular disease and normal cardiac troponin levels, 37.5% cardiovascular disease but elevated cardiac troponin levels, and 69.4% patients having both the conditions. A higher proportion of men (65.4%) had increased cardiac troponin as compared to women (42.2%) with COVID-19 (20).

The effect of estrogenic hormones could justify these observations, as this hormone has been reported to reduce low-density lipoprotein cholesterol and increase the high-density lipoprotein (21). 17β-estradiol, an estrogenic hormone, is also known to mediate the activation of early and late endothelial nitric oxide synthase *via* estrogen receptor interaction (22). Cardiomyocytes also carry the functional estrogen receptors that regulate the expression of nitric oxide synthase to prevent the cardiovascular system from damage by some factors such as suppression of the formation of thrombus, platelet stimulation, and adhesion of leukocyte-endothelial cell. It has been reported that male mice are more vulnerable to SARS-CoV compared to females. However, when the ovaries (an endocrine gland producing and releasing estrogen) from female mice were removed, their mortality from the SARS-CoV sharply increased (23).

**Figure 2:**
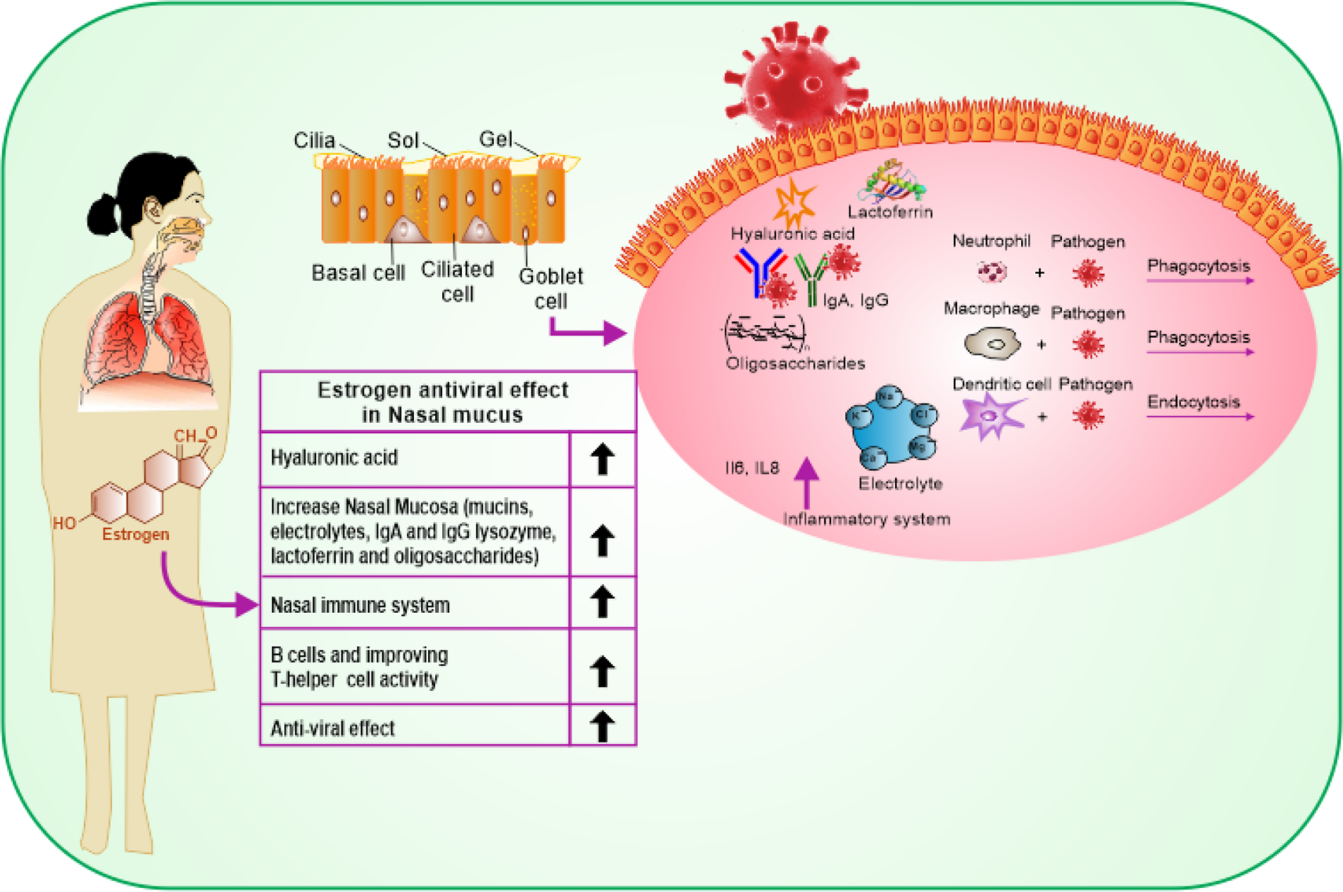
Schematic representation showing the protective effects of estrogen on the upper and lower respiratory tract cells and its benefits on the immune response.

COVID-19 affects men and women differently likely due to the difference in genetic nature and influence of sex hormones. COVID-19 enters the host body *via* the upper respiratory system, through contacting droplets. Estrogen has a beneficial impact on the entire respiratory tract system (16). Estrogen activates the response of mucosa of the nose by regulating turbinate hypertrophy and boosting secretion of nasal mucus containing anti-viral, antibacterial, and immune factors such as IgA, lysozyme, mucins, lactoferrin, electrolytes, and oligosaccharides, which are important for restricting upper airway infections (24). Besides, estrogen stimulates the synthesis of hyaluronic acid that preserves a suitable tropism of the cilia and the mucosal membrane (**Figure 2**). Additionally, estrogen stimulates the local nasal immune system that acts directly by stimulating phagocytic cells, antigen-presenting cells, and natural killer cells (25). Once stimulated, they can kill the virus protecting the body before its access to its target cells in the part of the respiratory system, thus reducing the pathological effect of the virus (26). In a study, it was indicated that G protein-coupled estrogen receptor (GPER) specifically supports the diminishing nasal symptoms, serum OVA-specific IgE, and Th2 cell immune response, but boosts the Treg immune response in mice (27). In addition to its indigenous impact in the nasal cavity, estrogen provides the required level of hyaluronic acid secretion needed for the mouth’s hydration by promoting the function of the lower respiratory system as it acts directly on the bronchial epithelial membrane to secrete more mucus. At this stage, the effective role of estrogen is promoted by the progesterone (PG) physiological function as it upregulates amphiregulin (epidermal growth factor) to maintain the histological integrity of the lung tissue if the viral infection occurs. PG also improves the onset of the symptoms of respiratory disease, when given to women at menopause phase (28). Estradiol (E2) and PG support a reduced case of a naive immune-inflammatory reaction, *via* increasing the immune tolerance and synthesis of immunoglobulins. It has been reported that the combination of E2 and PG could enhance the anti-viral immunity, but downplay cytokine storms in COVID-19 (29).

E2 has been found to have a protective activity against the disease severity, as revealed by higher levels of cytokines such as IL-6 and IL-8 in severe cases. E2 corresponded to COVID-19 severity, because of the regulation of such cytokines associated with inflammation (30). Also, regulatory proteins (Cardiac troponin T and troponin I) play a key role in calcium regulation (7).

Estrogen has anti-inflammatory and anti-oxidative actions on the effectors of the renin-angiotensin system-like pro-oxidative LOX-1 and pro-inflammatory ICAM-1. Estrogen alters the homeostasis of the local RAS and offers protection in the atrial myocardium (31). Moreover, other studies have indicated the anti-viral activity of two selective estrogen receptor modulators against viral infection like Ebola. Primary differentiated human nasal epithelial cell cultures obtained from healthy men and women demonstrated the action of estrogenic receptors on the human cellular response to influenza A virus (IAV) infections. Nasal epithelial cells are the primary cell type infected with IAV, and these cultures allowed to investigate IAV infection and pathogenesis based on the sex and hormonal milieu of the donor cultures (32).

Menopause is an individual risk factor for COVID-19 as it causes a sudden reduction in estrogen levels which could minimize the risk difference between men and women, although the case studies have revealed that the gender disparity still exists in elderly people. In postmenopausal women, the ovaries produce estrone, the inactive form of estrogen, in high quantities. Additionally, estrogen is no longer the only endocrine factor in the postmenopausal stage. A number of extragonadal tissues such as adipose tissue, bone chondrocytes and osteoblasts, aortic and endothelium, vascular smooth muscle cells, skin, skeletal muscle, and several brain regions produce estrogen, to act locally as a paracrine and intracrine factor (33). Therefore, circulating estrogen levels explain its effect in menopausal women because estrogen escapes from local metabolism and gets into the main circulation (33, 34). It is still unclear if the estrogen circulation and expression in the local tissue play a part in the reduced COVID-19 mortality in menopausal women compared to age-matched men (35). Therefore, the role of estrogen is fascinating.

## Difference in Innate and Adaptive Immunity

Women show reduced susceptibility to viral infections due to their varying nature of innate immunity, hormones, and other factors associated with sex chromosomes. Sex-related hormones regulate the range of the immune responses distinctively in men and women (36). The estrogen and ER-α influence the activation and proliferation of T-lymphocytes and initiate elevation of IFN-γ level in Th1 lymphocytes. A gradual IFN reciprocation by mismatched dsRNA or exogenous IFN-α treatment has been found to inhibit SARS-CoV multiplication in the lungs of mice (37). Studies have reported that IFN-β and IFN-γ can significantly suppress the replication of SARS-CoV, and a symbiotic anti-SARS-CoV action was attained with the synthesis of the IFN-β and IFN-γ (38). As discussed earlier, treatment with estrogen suppresses the inflammatory response and reduces SARS-CoV load that leads to an increased survival in mice (23). Contrary to estrogen, testosterone have a general inhibitory action on the immune response, specifically to viral antigens (39). In a study, murine macrophage treatment with testosterone suppressed the nitrate oxidase synthetase (40).

Studies have shown suppressive effects of testosterone on the activation of dendritic cells, antigen presentation to T-lymphocytes, and initiation of immune response (41). Th1 cells have a crucial part to play in protection against viral infections by secreting IFN-γ (42). Androgens can influence the thymocyte response by suppressing the Th1 proliferation and reducing IFN-γ synthesis (43–45).

**Figure 3:**
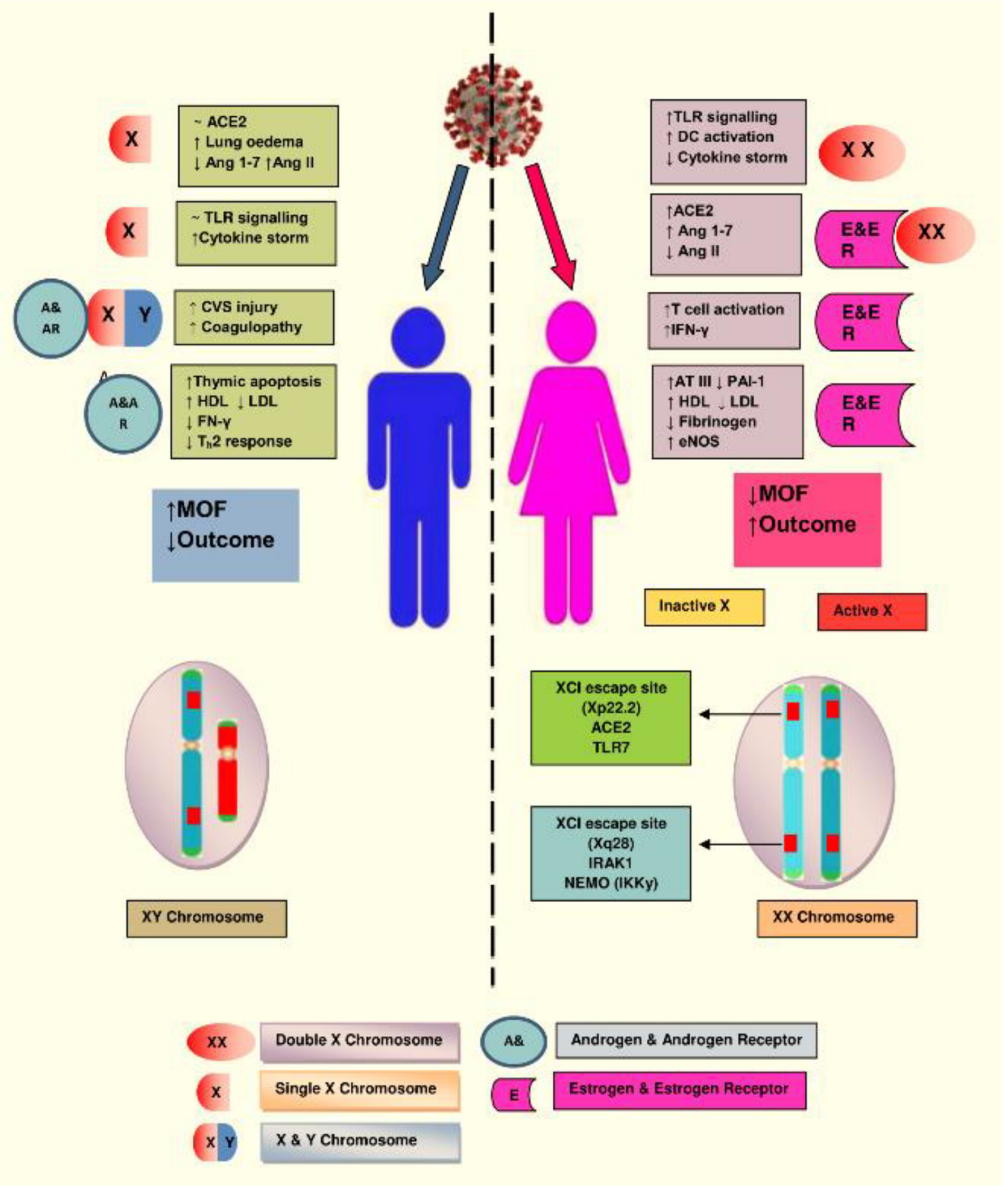
The X chromosome in females has various genes associated with immunity. Natural mutation in one copy of X gene may lead to two different alleles with distinct regulatory mechanism, which protect the women from implications of deleterious mutations and confers advantage in facing novel immunogens, like SARS-CoV-2. Illustration presents the genes encoded on the X-chromosome involved in the increased immune response in females during COVID-19. (Abbreviations used: TLR7, Toll-like receptor 7; DC, dendritic cell; CVS, cardiovascular system; IFN-γ, interferon gamma; LDL, low-density lipoprotein; HDL, high-density lipoprotein; ATIII, antithrombin III; IRAK1, Interleukin-1 receptor-associated kinase 1; MOF, multiorgan failure).

Among women, in order to reduce the duplication of X-linked genes, the second X chromosome is silenced *via* X chromosome inactivation (XCI), although many genes escape this inactivation. A location on Xp22.2, which is for the ACE2 gene, also bypasses X-inactivation, resulting in the phenotypic differences between the genders. The other XCI escaping regions are IRAK1 (Interleukin-1 receptor-associated kinase 1) and IKKγ (inhibitor of nuclear factor Kappa-B kinase subunit gamma) that might influence the immune response against the COVID-19 infection in women. Numerous genes are involved with the X chromosome. Mutations occurring in a single gene may lead to two different alleles with a distinct mechanism of response, suggesting that women could not only escape the outcome of deterrent mutations but also help to fight against infectious challenges such as SARS-CoV-2. Additionally, estrogen and estrogen receptor signaling confer an important potency to innate as well as adaptive immunity and the process of tissue repair during and after the viral infection (7, 36, 39) (**Figure 3**).

In SARS-CoV-2 infected women, T cells, especially CD8^+^ T cells, were found much more activated. When their clinical trajectory was analyzed, it was revealed that elevated cytokine levels in women patients were related to the worsening condition of COVID-19 disease (36). Most COVID-19 affected patients have higher plasma levels of pro-inflammatory cytokines/chemokines (IL-6, IL-2, IL-8, IL-7, CCL2, CCL3, and TNF) (3). This may lead to tissue damage and subsequent organ failure. Elevated levels of plasma cytokines are correlated with a decrease in lymphocytes which leads to the progression of COVID-19 disease. Dysfunction of T cells with age is also associated with worse COVID-19 disease outcomes (46). However, even though elderly women develop a strong T-cell immune response, majority develop anti-Spike IgG at the initial stage of infection that helps in suppressing proinflammatory cytokines, and hence, worsening of disease does not occur (47).

It is equally pertinent to mention that women are at no lesser risk of getting infected with coronavirus, especially during pregnancy, as women fall at higher risk of severe illness from other respiratory infections. COVID-19 infection in pregnant women did not differ much from non-pregnant women (48). Reports have suggested that pregnancy and childbirth do not seem to contribute to an increased risk of contracting SARS-CoV-2 infection; it also does not increase the severity of the clinical course of COVID-19, compared to non-pregnant women of the same age (49–51).

COVID-19 infection during pregnancy may have more unfavorable results in comparison with the non-pregnant group (52). Additionally, COVID-19 and pregnancy increase the chance of internal clotting that increases the risk of thrombosis (53). During the period of pregnancy, a large variety of immune cells, mostly natural killer (NK) cells, macrophages, and regulatory T cells (Treg), are activated. The accumulation of macrophages and NK cells takes place around trophoblastic cells during the first trimester of pregnancy protecting miscarriage of the allogeneic fetus (19). Hence, the maternal immune system shields fetus from the damage by environmental insults. Likewise, the fetus also modifies the maternal immune system. During pregnancy, PG has immunomodulatory effects that influence the Th1 response. In pregnancy, an enhancement in anti-inflammatory factors like interleukin-1 receptor antagonist (IL-RA) and TNF-α receptor (TNF-R) is recorded; conversely, a decrease in IL-1β and TNF-α is found (20).

Variations in the estrogen and PG levels during pregnancy may cause respiratory, cardiovascular, reversible degeneration in the thymus, with a reduction in CD4^+^ and CD8^+^ T cells that may lead to more susceptibility of women to SARS-CoV-2 infection. The PG on nasal mucosa acts as a facilitator in the attachment of the virus and prevents its elimination. Additionally, an increase in oxygen consumption due to vascular congestion and reduction in the capacity of the lung may increase the risk for severity of COVID-19 in pregnant women (54). Another risk factor is the higher ACE2 expression during pregnancy, and hence increased risk of complications from COVID-19 infection (55). An increase in ACE2 receptors in the kidneys during pregnancy may contribute to effective regulation of blood pressure, although it can favor the attachment and facilitate the virus entry into the host cells (54).

Androgens might lead to severe COVID-19 disease among men through raising neutrophil count and increasing the production of cytokines (IL-1β, IL-10, IL-2), altering TGF-β production by immune cells, and decreasing the antibody production (47). This event is crucial as the patients with severe COVID-19 exhibit cytokine storm syndrome due to neutrophils. One of the androgen pathways in COVID-19 infection is the transmembrane protease, serine 2 (TMPRSS2) gene that is expressed mainly in the adult prostate (56), and in metastatic prostate cancers; it is also found in tissues like lung, kidney, pancreas, colon, small intestine, and liver (56). The TMPRSS2 gene is transcribed and regulated by the androgen receptor, and the main target of TMPRSS2 expression in COVID-19 is the lungs, kidneys, and liver (57). In one retrospective study, increased levels of testosterone in most women (60%) having COVID-19 disease were recorded; a positive correlation between the levels of testosterone and pro-inflammatory cytokines among women with COVID-19 was also noted (58).

In view of a higher mortality in men from COVID-19 compared to women, it has recently been pointed out that testosterone may affect disease severity. This notion is supported by the evidence that the primer protease for SARS-CoV-2 spike protein, TIMPRSS2, as well as the virus entry receptor, ACE2, are upregulated by testosterone (59). Although debated, androgen-deprivation therapy in prostate cancer patients infected with SARS-CoV-2 has been suggested (60). However, hypogonadism can also be a risk factor for severe COVID-19 (61). It is worth noting that women suffering from polycystic ovarian syndrome (PCOS), which is characterised by heightened androgen levels (hyperandrogenism), have been found to be at a significantly higher risk of COVID-19 compared to non-PCOS women (62, 63).

## Role of Angiotensin-Converting Enzyme 2 (ACE2)

The ACE2 gene that is found on the X chromosome (location: Xp22.2; nucleotides 15 494 402–15 602 148, GRCh38.hg38 version) has been reported to work differently in men and women (64). ACE2 carries out its important functions by dissociating angiotensin I into angiotensin II. Angiotensin II, being a small peptide, is of huge importance in the case of vasoconstriction and sodium balance. ACE2 breaks angiotensin I and II into dissociated peptides that possibly lead to vasodilatation and, hence, countering angiotensin II (65, 66). The entry route for SARS-CoV-2 is *via* ACE2, similar to the SARS-CoV virus, bearing a spike protein that binds with ACE2 to invade the cells (20, 46, 67). The location of the ACE2 gene on Xp22.2 is a site of genes that escapes XCI, leading to phenotypic dissimilarities between genders (68, 69). SARS-CoV-2 possesses 16 residues of receptor binding motif (RBM), and binds to 16 of the 20 ACE2 residues present in men. In women, the same RBM of SARS-CoV-2 may be detected by ACE2 on any of the two X chromosomes. The possibility becomes less for the similar residue sequences of ACE2 present on the second chromosome to bind efficiently to the RBM of SARS-CoV-2, leading to the breakdown of Ang II to form Ang 1–7 by unbound ACE2, and therefore might reduce the chance of respiratory edema during SARS-CoV-2 infection. Men, with only one X chromosome, are deficient in the alternative mechanisms that could impart cellular protection during COVID-19 infection (70, 71).

Several significant divergences in the prevalence of ACE2 variants have been reported among diverse races and ethnicity. Recently, a single-cell RNA sequencing (RNA-seq) study reported that Asian men could express tissue ACE2 at a higher level (72). During a study on the northeastern Chinese Han population, the serum ACE2 activity was found to have a negative correlation with body mass index, pulse pressure, and estrogen levels among hypertensive women (6). Such studies indicate a protective mechanism of circulating ACE2 and the participation of estrogens in the expression and upregulation of ACE2 activity levels (73).

ACE2 is present in epithelial cells of the lung, intestine, blood vessels, and kidney (74). The angiotensin system plays a vital role in cardiovascular homeostasis, acute inflammation, and autoimmune disorders (75). The presence of high ACE2 receptors may lead to a higher risk of contracting SARS-CoV2. It has been reported that men have elevated levels of circulating ACE2 than women, and also in patients having diabetes and cardiovascular ailments (76). People with cardiovascular failure have the plasma ACE2 elevated in men compared to women, which correlates with increased SARS-CoV infection (65, 77). Among the hypertensive women, blood pressure and body mass index inversely correspond to ACE2, whereas there is a direct correlation of blood sugar and estrogen levels to ACE2 level (65, 78). As mentioned above, estrogen also downregulates the renin-angiotensin system components acting as an anti-inflammatory and anti-oxidative agent (67, 78). Significant functional regulation of ACE2 by estrogen may explain the gender differences in COVID-19 associated morbidity and mortality (79).

### Microbiota

Development of a pronounced innate and adaptive immune response is greatly influenced by the composition of the human gut microbiota. The human gut possesses a diverse and complex microbial consortium that reciprocates by establishing the persistent host immune homeostasis (80–82). The human gut harbors complex communities of microorganisms that includes holobiont (composite organism) and hologenome (collective genome of all bionts) (83). This complex composition offers a crucial genomic and metabolic capability that has an important impact on the initiation, development, and action of the host immune system, thereby protecting against infections and safeguarding the ecosystem of gut flora (84). The homeostatic cascades existing between the immune system and gut microbiota of the host play a crucial role in modulating the activation of host cells and tissues involved in response to infectious agents (85). The interaction of virus and microbiota has been studied in several viral infections. For example, surfactin, a molecule on a *Bacillus subtilis* surface, is known to disintegrate many viruses including influenza A (85). Thus, the gut microbiota is likely to influence COVID-19 pathogenicity, and conversely, SARS-CoV-2 may influence the gut microbiota leading to dysbiosis and other unpleasant consequences (86). Therefore, the alteration of the composition of existing microbiota and health conditions during SARS-CoV-2 infection is likely to have a major role in establishing the susceptibility and resilience of an individual to COVID-19. However, most of the COVID-19 severe symptoms and fatalities occur in individuals having some risk factors such as aging, preexisting comorbidities, and, to some extent, gender, which are indirectly characterized by disrupted microbiome status (87).

**Figure 4:**
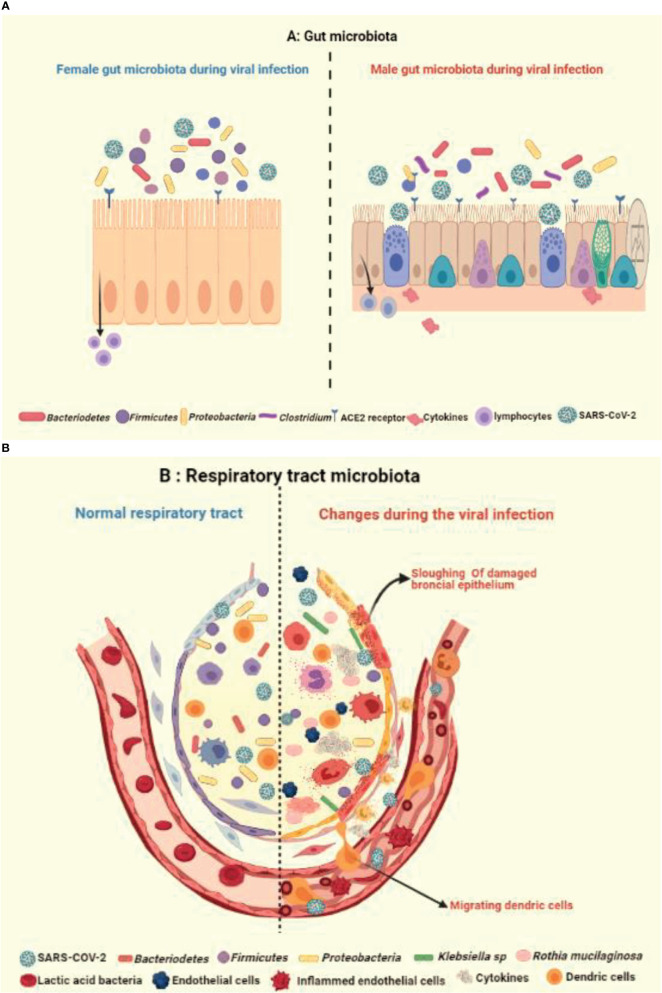
Illustration showing the impact of healthy and unhealthy microbiota on respiratory tract infection. The complex relationship *via* gut-lung axis might be crucial in determining the vulnerability of respiratory tract to COVID-19, as an outcome of potential variation and crosstalk between **(A)** healthy gut microbiota with occurrence of fewer *Bacteriodetes* and **(B)** respiratory microbiota with prevalence of more *Klebsiella* and *Ruthia sps* in virus infected alveolus.

Like gastrointestinal system, the respiratory microbiome constitutes the community of differentiated bacterial phyla like *Bacteroidetes*, *Firmicutes*, and *Proteobacteria* and has a protective role in the host immunity (44, 88) (**Figure 4A**). Han et al. showed that the COVID-19 infection can alter the lung microbiome (89). A severe dysbiosis was found among COVID-19 patients, with a higher prevalence of pathogenic microbes such as *Klebsiella oxytoca*, *Faecalibacterium prausnitzii*, *Lactic Acid Bacteria*, and *Tobacco mosaic virus* (TMV) (**Figure 4B**). The serious inflammatory environment in the lungs correlated with *Rothia mucilaginosa*, TMV level, and SARS-CoV-2, suggesting a key role of respiratory microbiota in COVID-19 disease. Other studies also reported fecal microbial changes in 15 subjects infected with COVID-19 that correlated with high severity and abundance of *Coprobacillus*, *Clostridium ramosum*, and *Clostridium hathewayi*, and reduced levels of *Faecalibacterium prausnitzii* and *Alistipes onderdonkii* (90, 91) (**Figures 4A, B**).

The microbiota existing outside of the reproductive tract is significantly mediated by the sex steroid hormones. Many studies conducted on mice, fish, and humans have analyzed the sex difference in gut microbiota. This subject of whether the sex difference in gut microbiome in humans has any involvement in the disparity of viral infection is an interesting area to study (92). In a study, gender differences correlated with the overall composition of gut microbiota. The gut microbiome in women was found to have a lower occurrence of *Bacteroidetes* compared to men (93). An animal study evaluated gender-specific variations in the composition of gut microbiota (94, 95). The systemic estrogen levels may be influenced by dietary fiber, which is the main energy source of gut microbial fermentation and, hence, formulates the gut microbiota (94, 96).

## Environmental Mediators

In addition to biological differences accounting for a significant gender disparity of COVID-19, the influence of environmental factors could also play a part (97).

### Lifestyle

Lifestyle choices among the genders possibly makes a huge difference. Historically, it has been noticed that men are more habitual of smoking than women. Smokers tend to have weakened lungs leading to chronic lung and heart diseases that could be the worst outcome, if infected with COVID-19 (97–100). In China, the smoking prevalence in men is 57.6% which is nearly 10 times more than the women with 6.7% (101). The lower airways of smokers have shown a higher expression of ACE2, suggesting a higher risk for COVID-19 (102, 103). Such findings are an indication of one of the factors behind the increased mortality in men with COVID-19 which needs further validation.

### Exercise

The decreased incidence rate of COVID-19 symptoms in women can be also related to the physical activity engaging from moderate-to-vigorous one. Women are considered to be physically more active when compared with men who prefer prolonged and intensive exercises (1, 104). Prolonged and vigorous exercise may lead to immunosuppression; on the contrary, mild and moderate exercise enhances immune response and significantly minimizes the risk and severity of respiratory viral infection. This is supported by a number of studies that explain a moderate level of exercise lowers inflammation and boosts the immune function. Regular mild physical activity influences the level of hormones related to stress, which downregulates intense inflammation of the respiratory tract and helps in activating the anti-viral innate immunity polarising the immune function towards a Th2 profile, extensive research is needed to study cellular and molecular cascades through which exercise regulates immune response (104–106).

### Nutrition

A study shows nutritional environment during the post and prenatal period is associated with a reduced mortality rate among females in case of HIV, for example, high-fat diet and the micronutrients like Vitamin B, C, and E supplements have a reduction of 32% (107). Another study suggests the benefits of supplementary maternal micronutrients in women compared to men (108, 109).

## Conclusions and Perspective

Immunity, X-chromosome associated genes, and sex hormones are the main distinguishing factors that are likely to offer greater resistance against SARS-CoV-2 in women. The evidence suggesting important decisive factors of gender-related disparity in immunity may impact on the onset of COVID-19 and vaccination outcomes.

## Author Contributions

SR: conceptualized, drafted, figures, and revised. VN contributed to figure, environmental factors, and reference formatting. IN: contributed to epidemiology part. HA: contributed to environmental part. MS: contributed to mortality and map part. MA contributed to severity and manifestation part. UK: reviewed, edited, and revised the manuscript. EAl-S: conceptualized, drafted, revised, and edited. All authors read the article and approved the submitted version.

## Funding

Institute for Research and Medical Consultation (IRMC) is highly acknowledged for the research facilities.

## Conflict of Interest

The authors declare that the research was conducted in the absence of any commercial or financial relationships that could be construed as a potential conflict of interest.

## Publisher’s Note

All claims expressed in this article are solely those of the authors and do not necessarily represent those of their affiliated organizations, or those of the publisher, the editors and the reviewers. Any product that may be evaluated in this article, or claim that may be made by its manufacturer, is not guaranteed or endorsed by the publisher.
